# The effect of traditional diet on glucose homoeostasis in carriers and non-carriers of a common *TBC1D4* variant in Greenlandic Inuit: a randomised crossover study

**DOI:** 10.1017/S000711452300106X

**Published:** 2023-12-14

**Authors:** Jack Ivor Lewis, Mads Vendelbo Lind, Grith Møller, Torben Hansen, Hanne Pedersen, Marie Mathilde Bjerg Christensen, Jens Christian Laursen, Sara Nielsen, Charlotte B. Ottendahl, Christina V. Lytken Larsen, Ken D. Stark, Peter Bjerregaard, Marit E. Jørgensen, Lotte Lauritzen

**Affiliations:** 1 Department of Nutrition, Exercise and Sports, Faculty of Science, University of Copenhagen, Copenhagen, Denmark; 2 The Novo Nordisk Foundation Center for Basic Metabolic Research, Faculty of Health and Medical Sciences, University of Copenhagen, Copenhagen, Denmark; 3 Steno Diabetes Center Copenhagen, Gentofte, Denmark; 4 National Institute of Public Health, University of Southern Denmark, Odense, Denmark; 5 Department of Kinesiology and Health Sciences, University of Waterloo, Waterloo, Canada; 6 SDU, Copenhagen, Denmark; 7 Ilisimatusarfik, The University of Greenland, Nuuk, Greenland

**Keywords:** Marine diet, TBC1D4 genotype/p.Arg684Ter, Insulin sensitivity, Cardio-metabolic markers, *n*-3 LCPUFA

## Abstract

Consumption of traditional foods is decreasing amid a lifestyle transition in Greenland as incidence of type 2 diabetes (T2D) increases. In homozygous carriers of a *TBC1D4* variant, conferring postprandial insulin resistance, the risk of T2D is markedly higher. We investigated the effects of traditional marine diets on glucose homoeostasis and cardio-metabolic health in Greenlandic Inuit carriers and non-carriers of the variant in a randomised crossover study consisting of two 4-week dietary interventions: Traditional (marine-based, low-carbohydrate) and Western (high in imported meats and carbohydrates). Oral glucose tolerance test (OGTT, 2-h), 14-d continuous glucose and cardio-metabolic markers were assessed to investigate the effect of diet and genotype. Compared with the Western diet, the Traditional diet reduced mean and maximum daily blood glucose by 0·17 mmol/l (95 % CI 0·05, 0·29; *P* = 0·006) and 0·26 mmol/l (95 % CI 0·06, 0·46; *P* = 0·010), respectively, with dose-dependency. Furthermore, it gave rise to a weight loss of 0·5 kg (95 % CI; 0·09, 0·90; *P* = 0·016) relative to the Western diet and 4 % (95 % CI 1, 9; *P* = 0·018) lower LDL:HDL-cholesterol, which after adjustment for weight loss appeared to be driven by HDL elevation (0·09 mmol/l (0·03, 0·15), *P* = 0·006). A diet–gene interaction was indicated on insulin sensitivity in the OGTT (p = 0·093), which reflected a non-significant increase of 1·4 (–0·6, 3·5) mmol/l in carrier 2-h glucose. A Traditional diet marginally improved daily glycaemic control and plasma lipid profile compared with a Westernised diet in Greenlandic Inuit. Possible adverse effects on glucose tolerance in carriers of the *TBC1D4* variant warrant further studies.

The Greenland Inuit have experienced a marked increase in type 2 diabetes (T2D) prevalence to almost 10 % in recent surveys^(1,2)^. The rising T2D epidemic may be driven by social and cultural transitions of Greenlandic Inuit initiating a dietary shift from predominantly locally available marine foods towards increasingly Westernised imported foods^(3)^.

Genetic predisposition may exacerbate the risks of this transition^(4)^. A recent study among Greenland Inuit identified a nonsense variant (p.Arg684Ter) in *TBC1D4*, strongly associated with T2D risk^(5)^. The *TBC1D4*-encoded protein is required for insulin-dependent translocation of GLUT4 to the plasma membrane for glucose uptake^(6)^. Homozygous carriers of the p.Arg684Ter variant present with postprandial insulin resistance of skeletal muscle resulting in impaired glucose tolerance (IGT) and hyperglycaemia following a glucose challenge. The variant may increase susceptibility to modifiable risk factors associated with the ongoing lifestyle transition in modern Greenland.

Traditional Inuit diets, rich in fish and marine mammals, result in high proportions of energy consumed (E%) as protein and fat, as well as a high intake of *n*-3 long-chain PUFA (LCPUFA)^(7)^. Reduced intake of these LCPUFA may contribute to impaired glycaemic control, as *n*-3 LCPUFA status has been associated with improved insulin sensitivity and lower fasting glucose^(8)^ and a recent meta-analysis found that substitution of SFA with PUFA may reduce glycated haemoglobin (HbA1c) and insulin resistance assessed by the homoeostasis model (HOMA-IR)^(9)^. Increased consumption of simple sugars and refined grains in the modern Inuit diet may further contribute to T2D risk^(10)^ via effects on the glycaemic index^(11)^. Elevated blood glucose clusters with other cardio-metabolic risk factors including abdominal adiposity, dyslipidaemia and high blood pressure (BP), which may also be affected by these dietary components. Randomised clinical trials (RCT) in Western populations have consistently shown that *n*-3 LCPUFA increase HDL-cholesterol and reduce TAG^(12)^, BP^(13)^ and C-reactive protein (CRP)^(14)^, whereas SFA are positively associated with LDL-cholesterol^(15)^ and simple carbohydrates with plasma TAG^(16)^.

The aim of this crossover RCT was to investigate whether a Traditional marine diet can improve glycaemic control in Greenland Inuit. The hypothesis was that a 4-week Traditional diet would result in a greater reduction in 2-h plasma glucose in an oral glucose tolerance test (OGTT, primary outcome) compared with after a 4-week Western diet. We studied additional markers of glucose homoeostasis (secondary outcomes) and if any effect is modified by the *TBC1D4* p.Arg684Ter variant. Changes in cardio-metabolic health markers were also compared between the two diets.

## Methods

This randomised crossover study was conducted from 2019 to 2020 at three sites located in the towns of Nuuk, Qaanaaq and Qasigiannguit, Greenland.

### Ethical approval

This study was conducted according to the guidelines laid down in the Declaration of Helsinki, and all procedures involving human subjects were approved by the Ethics Committees of Greenland (KVUG 2018-26) and Denmark (H-18055643). Written consent was obtained from participants after receiving written and oral study information in Greenlandic or Danish, as appropriate. The study was registered at ClinicalTrials.gov (NCT04011904), although after enrolment began due to administrative error, and the study protocol has been published^(17)^.

### Participants

Homozygous carriers or homozygous non-carriers of the *TBC1D4* p.Arg684Ter variant were invited to participate based on a register of enrolment in two previous studies^(18,19)^. All were of Inuit descent, defined as both parents and four grandparents being born in Greenland. Individuals were contacted with study information in various ways: in Nuuk (64·1814° N, population: 18 800) by letter and telephone, in Qasigiannguit (68·8166° N, population: 1081) by telephone and in Qaanaaq (77·4669° N, population: 646) by letters and house visits. Those interested were invited for a screening visit.

Following informed consent, participants were screened according to inclusion and exclusion criteria (see protocol article^(17)^). Briefly, they were required to be aged 18–80 years and speak Greenlandic and/or Danish. Participants were excluded if they had BMI ≤ 18·5 kg/m^2^, had previously been diagnosed with diabetes or their baseline HbA1c measured ≥ 48 mmol/mol (6·5 %) or were receiving pharmacological treatment which may interfere with the study. One participant with a BMI of 16·1 kg/m^2^ was mistakenly recruited, but a decision was taken to keep them in the study.

### Randomisation and blinding

After baseline measurements, enrolled participants were randomised to receive either a ‘Traditional’ or ‘Western’ dietary intervention first. Block randomisations of random block sizes were performed by a statistician not related to the project using R software (version 3.5.3) and the *blocrand* package. Randomisation lists were generated for each site, which in Nuuk and Qaanaaq were stratified by genotype. Participants were given an ID based on the order of their first visit, which determined their allocation to diet intervention sequence. Blinding of participants and investigators was not feasible during the study, but blinding was maintained during sample and initial data analysis such that researchers knew the period but not sequence allocation.

### Dietary intervention

Participants received two 4-week dietary interventions. During the Traditional dietary period, foods consisted mostly of marine mammals, fish and reindeer while targeting a high fat content (> 40 % of the energy intake (E%)) and low carbohydrate content (< 30 E%), with limited grains and imported foods permitted. For the Western diet, we targeted a high carbohydrate (55–65 E%) and moderate fat (30–35 E%) content, with marine fats contributing little and saturated fats from imported meats more so. A proportion of the diet (> 20 E%) was provided to participants, depending on local and seasonal availability.

In the Traditional dietary period, participants received fish, seafood, sea mammals and game. In the Western dietary period, they received imported meats, breads, pasta, rice and cereals. For the remaining dietary intake, participants were provided with basic written instructions encouraging adherence to the appropriate dietary pattern. For further details on the diets, refer to the protocol article^(17)^. In both periods, participants were asked to limit alcohol intake and maintain a basic food log to assess dietary adherence.

Data collection took place at baseline and after each of the two intervention periods.

### Interviews and dietary assessment

Baseline information was collected with an interviewer-led questionnaire conducted in the participant’s language of choice. A household asset score (0–10) was derived from the number of seven items (microwave, computer, etc.) found in the home, modified for Greenland Inuit^(20)^. Data on smoking status, alcohol consumption and medication were also collected. Physical activity energy expenditure was estimated at baseline and for each intervention period using a modified long version International Physical Activity Questionnaire (The IPAQ Group, Sweden), validated in the Greenlandic population^(21)^.

Baseline habitual food intake was assessed with a validated semi-quantitative FFQ^(22)^, which was also used to assess intake in the intervention periods as a measure of compliance. The FFQ assessed frequency and portion size of common traditional and imported foods during the previous month. Portion sizes were estimated from four food-specific portions shown with photographs, and estimates of intake in grams per day (g/d) were then derived for each period. Energy, macronutrient and *n*-3 LCPUFA estimates were calculated based on published nutrient content (Danish Food Composition Databank, National Food Institute, Denmark)^(23)^, after removing six FFQ entries with unrealistic daily intakes (< 3350 kJ and < 2100 kJ for men and women, respectively, and > 17000 kJ and > 15000 kJ for men and women, respectively).

### Clinical measures

BP was measured three times during the interview using a mercury sphygmomanometer with an appropriate size cuff and an average the two lowest readings was used for further analysis. Height was measured without footwear to the nearest cm using a stadiometer (Chasmore) and standing waist (at the navel) and hip circumference measured in duplicate to the nearest 0·5 cm. Weight was measured to the nearest 0·1 kg with heavy clothing removed using a Tanita TBF-300MA (Tanita Corporation) scale. Body fat percentage and fat-free mass were measured to the nearest 0·1 % and 0·1 kg, respectively, using the same Tanita scale, which functioned as a bioelectrical impedance analyser using internal estimating equations based on default body type.

A Freestyle Libre Pro (Abbott Diabetes Care) 24-h continuous glucose monitor (CGM) was fitted to each participant at the end of the baseline and second visit to assess blood glucose for the first 14 d of each intervention period. If the monitors detached within 9 d, they were equipped with a second monitor and data combined.

At each study visit, fasted blood samples were first taken by venipuncture. HbA1c was analysed immediately by a DCA Vantage Analyser (Siemens Healthcare). Further blood samples were collected into sodium fluoride-EDTA-citrate tubes, dry tubes and EDTA tubes. Centrifuged sodium fluoride-EDTA-citrate tubes were used for glucose analysis, and serum was separated in dry tubes for insulin, blood lipids and CRP analysis. Aliquots of whole blood from EDTA tubes were stored with 5 µl 0·01 % butylated hydroxytoluene per ml for the analysis of fatty acid composition.

Fasting blood samples were followed by an OGTT. Participants consumed 247 ml (333 mg/ml) of glucose monohydrate equivalent to 75 g of glucose within 5 min. Postprandial blood samples were taken after 30 and 120 min for insulin and glucose measurements. All samples were stored at −20°C at each study site for the remainder of that site’s study term (up to 12 weeks), before being transported to Denmark where they were stored at −80°C prior to analysis at Department of Nutrition, Exercise and Sports. Glucose, cholesterol, TAG and high sensitivity CRP were measured on a Horiba ABX Pentra 400 and insulin was measured on a Siemens IMMULITE 2000.

Insulin sensitivity was assessed for each visit using three indices: HOMA-IR, insulin sensitivity index (ISI_0·120_)^(24)^ and BIGTT-S_i_^(25)^. A conversion factor of 7·127 was used to convert insulin pmol/l to mU/l. Pancreatic *β*-cell function was calculated using BIGTT-AIR^(25)^.

In processing CGM data, the first incomplete day was removed from the dataset to account for diurnal fluctuations in blood glucose. Mean, minimum, maximum and range (maximum – minimum) of glucose values were calculated for each 24-h period. For each of these measures, the mean average across all recorded days of that intervention period (a median of 14 d (IQR 12–14) in both periods) was used for further analyses. The WHO criteria were used to classify participants as having impaired fasting glucose (fasting plasma glucose ≥ 6·1 to ≤ 6·9 mmol/l), IGT (fasting plasma glucose < 7 mmol/l and 2-h plasma glucose ≥ 7·8 to < 11·1 mmol/l) or diabetes (2-h plasma glucose ≥ 11·1 mmol/l or fasting plasma glucose ≥ 7 mmol/l). Results from the physical activity questionnaire were processed according to standard procedures. Participants were processed regardless of age (> 69 years, *n* 6), but the scores of two participants were removed for reporting > 960 min/d physical activity and any reports of > 180 min in each intensity domain of physical activity (walking, moderate, vigorous) were truncated to 180 min.

Whole-blood fatty acid composition was analysed at the Department of Kinesiology and Health Sciences, University of Waterloo, Canada as previously described^(26)^. In brief, the samples were trans-esterified directly with 14 % boron trifluoride and methanol and 22:3*n*-3 methyl ester (Nu Chek Prep Inc.) was added as an internal standard to the final hexane extract. The trans-methylated fatty acids were separated by fast gas chromatography on a Varian 3900 gas chromatograph with a DB-FFAP capillary column (15 m × 0·10 mm diameter × 0·10 μm film thickness, J&W Scientific from Agilent Technologies). Peaks were identified by retention time comparisons with an external standard (GLC-462; Nu Chek Prep Inc.), and fatty acids were expressed as relative weight% of the total fatty acids. The percentage of *n*-3 LCPUFA in total LCPUFA ((20:3*n*-3 + 20:5*n*-3 + 22:5*n*-3 + 22:6*n*-3)/(20:3*n*-3 + 20:5*n*-3 + 22:5*n*-3 + 22:6*n*-3 + 20:3*n*-6 + 20:4*n*-6 + 22:4*n*-6 + 22:5*n*-6 + 20:3*n*-9) × 100) was also calculated as it has previously been shown to be a robust biomarker of *n*-3 PUFA status for field studies where challenges in sample handling increase the risks of PUFA oxidation^(27,28)^. The mean concentration of fatty acids in the samples was 2·1 (sd 0·6) μg/mg, and we identified 92·5 (sd 1·7) % of the total fatty acid peak area.

### Statistical analysis

Our power calculation was based on 80 % power to detect a 1·1 mmol/l change in 2-h glucose between the diet periods (primary outcome) or an equivalent 0·6 × sd change within carriers and 0·4 × sd change in metabolic traits of the whole group. With a 15 % dropout rate, thirty carriers and thirty non-carriers were required^(17)^.

Baseline comparisons were made using Student’s *t* tests and Kruskal–Wallace rank sum tests for normally and non-normally distributed data, respectively. Pearson’s *χ* ^2^ tests were used for categorical data. Reported dietary intake from food logs and FFQ was compared using Pearson’s correlation coefficients. Marine mammal, fish, imported meats and cereal intake (g/d) were all compared with the same categories from the food logs. The ‘traditional meat’ category of the FFQ was compared with the ‘local specialty’ category of the food logs, which includes reindeer, musk oxen and ground birds as examples.

Testing for diet effect used complete case analysis, with data required from all three visits (two for CGM) to qualify for inclusion. The effect of diet on the primary outcome (2-h glucose) and secondary outcomes (fasting glucose and insulin, 2-h insulin, HOMA-IR, BIGTT-AIR, BIGTT-S_i_ and ISI_0,120_) was assessed with linear mixed models with fixed (diet, baseline value (where available), order and visit) and random effects (participant, site). Stratified analyses were specified for the glucose homoeostasis outcomes. Genotype effect modification was also examined by addition of a diet × genotype interaction term to test the effect of the *TBC1D4* variant. The same models were used for the cardio-metabolic risk factors, which also included a dichotomous proxy variable for medication; models for cholesterol and TAG were adjusted for lipid medication and those for BP were adjusted for hypertension medication. All models were checked by visual inspection of residual and normal probability plots. Logarithm and square-root transformations were used and the approximate method for back-transformation was used for presenting results^(29)^. Adjusted bar charts with standard errors were constructed for 2-h glucose using estimated marginal means derived from the linear mixed model and R package *emmeans*.

Two sets of sensitivity analysis were performed: one by adding a term for acute viral or bacterial infection (CRP > 10 mg/l) and another by adding a term for weight change calculated as the difference in weight since the prior visit.

All statistical analyses were performed with R software version 3.5.3 (The R Foundation for Statistical Computing, 2012) using the packages *lme4, ggplot2, arsenal, multcomp* and *emmeans.* Data are presented as mean ± sd, median (25th–75th percentile) or percentages as appropriate. A significance level of *P* = 0·05 was used throughout and *P* < 0·1 was taken as an indicator of potential effect modification, which was then examined by stratified analysis.

## Results

Of the sixty-three Inuit adults who were randomised in the study, eleven were homozygous carriers and fifty-two homozygous non-carriers of the *TBC1D4* variant. Baseline characteristics were comparable across the two genotypes (Table 1) and both allocations (online Supplementary Table 1) indicating a successful randomisation. The participants were heterogeneous, with a range in age and BMI from 36 to 79 years and from 16·1 to 40·8 kg/m^2^, 56 % were male and 84 % were characterised as having full Inuit ancestry. Most participants consumed alcohol ≤ 1 time per week and the median level of physical activity was high, but 59 % were current smokers. Their BP and lipid profile appeared healthy, but with high LDL-cholesterol levels, and according to the WHO criteria, fifteen participants (three carriers, twelve non-carriers) had impaired fasting glucose, nine (two carriers, seven non-carriers) had IGT, and a further five participants (all carriers) had diabetes according to a 2-h plasma glucose ≥ 11·1 mmol/l, one of whom also had an fasting plasma glucose ≥ 7 mmol/l. Health and lifestyle characteristics were similar across the sites (online Supplementary Table 2).

**Table 1.** Baseline characteristics of enrolled participants by genotype* (Mean values and standard deviations)

	Total (*n 63*)		Carriers (*n* 11)		Non-carriers (*n* 52)		
	Mean	sd	Mean	sd	Mean	sd	*P* †
Traditional – Western allocation							
*n*	33		6		27		0·874
%	52 %		55 %		52 %		
Female							
*n*	34		3		31		0·051
%	54 %		27 %		60 %		
Age (years)	56	10	56	12	57	10	0·906
Anthropometrics							
Height (cm)	164	9	166	10	164	9	0·457
Weight (kg)	75	17	74	21	75	17	0·955
BMI (kg/m^2^)	27·6	5·4	26·8	6·9	27·7	5·1	0·618
FFM (kg)	50	11	53	12	50	11	0·296
Body fat (%)	31	10	27	11	33	10	0·077
Waist circumference (cm)	100	14	97	17	101	13	0·363
Hip circumference (cm)	103	9	99	8	104	9	0·190
Cardio-metabolic risk factors							
Systolic blood pressure (mmHg)	121	16	121	16	121	17	0·978
Diastolic blood pressure (mmHg)	75	9	73	11	75	9	0·414
Pulse (bpm)	74	11	76	13	74	11	0·688
Total cholesterol (mmol/l)	5·90	1·07	5·87	0·97	5·90	1·10	0·927
HDL-cholesterol (mmol/l)	1·62	0·35	1·75	0·36	1·59	0·35	0·192
LDL-cholesterol (mmol/l)	3·91	1·00	3·66	0·79	3·97	1·04	0·363
TAG (mmol/l)							
Median	1·3		1·27		1·26		0·828
IQR	0·9, 1·6		0·90, 1·77		0·94, 1·61		
CRP (mg/l)							
Median	1·4		0·72		1·70		0·105
IQR	0·5, 2·9		0·28, 1·56		0·57, 3·27		
HbA1c (%)							
Median	5·5		5·70		5·50		0·298
IQR	5·4, 5·7		5·25, 6·05		5·40, 5·70		
	*n*	%	*n*	%	*n*	%	
Previous diagnosis							
Hypertension	26	42·6 %	5	45·5 %	21	42·0 %	0·834
Heart clot	4	6·3 %	0	0·0 %	4	7·7 %	0·342
Heart disease	9	14·5 %	1	9·1 %	8	15·7 %	0·573
Brain clot	6	9·5 %	0	0·0 %	6	11·5 %	0·236
Asthma	6	9·5 %	1	9·1 %	5	9·6 %	0·957
COPD	4	6·5 %	1	10·0 %	3	5·8 %	0·618
Recent antibiotics‡	7	12·5 %	1	11·1 %	6	12·8 %	0·284
Smoking status							
Current smoker	37	58·7 %	5	45·5 %	32	61·5 %	
Previous smoker	20	31·7 %	5	45·5 %	15	28·8 %	
Never smoked	6	9·4 %	1	9·1 %	5	9·6 %	0·551
Alcohol frequency							
≤ once per month	38	61·3 %	7	63·6 %	31	60·8 %	
2–4 times per month	19	30·6 %	2	18·2 %	17	33·3 %	
≥ 2 times per week	5	8·1 %	2	18·2 %	3	5·9 %	0·303
Physical activity (MET min ×10^3^)							
Median	5·0		5·9		4·8		0·541
IQR	3·0, 9·1		3·7, 10·2		2·8, 9·0		
Employment type							
Full time	39	61·9 %	8	72·7 %	31	59·6 %	
Self-employed	4	6·3 %	0	0·0 %	4	7·7 %	
Pensioner	11	17·5 %	1	9·1 %	10	19·2 %	
Other	9	14·3 %	2	18·2 %	7	13·5 %	0·620
Asset score							
Mean	7		7		7		0·387
sd	2		2		2		

COPD, chronic obstructive pulmonary disease; CRP, C-reactive protein; FFM, fat-free mass; HbA1c, glycated haemoglobin; MET, metabolic equivalents.

* Data presented as mean and sd for normally distributed continuous variables, median with IQR for non-normally distributed continuous variables and number and % for categorical variables.

† Level of significance for differences between genotype groups assessed by linear model ANOVA and Kruskal–Wallis rank sum test for normally and non-normally distributed continuous variables, respectively, and by Pearson’s *χ* ^2^ for categorical variables.

‡ In the last 3 months.

**Fig. 1.**
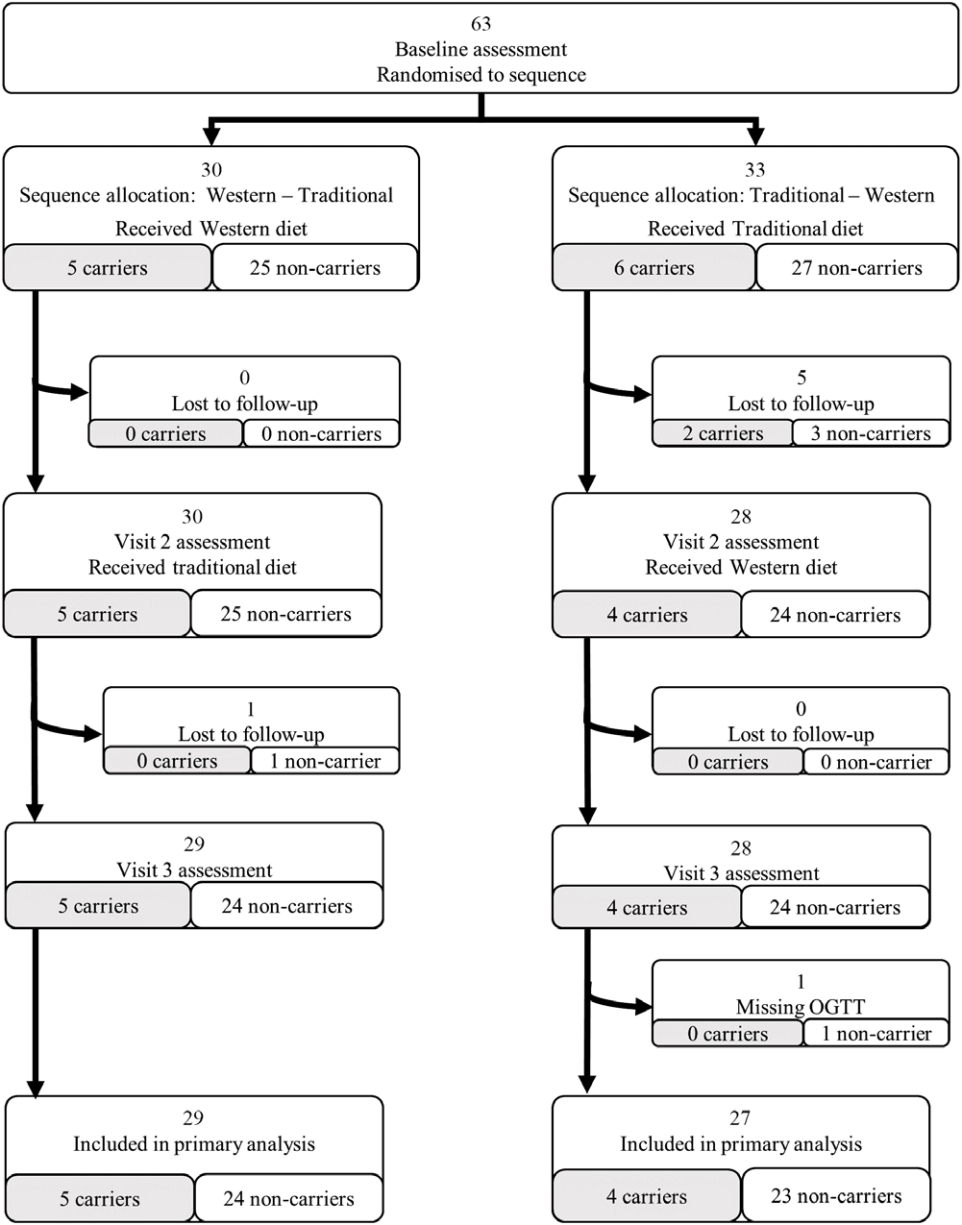
Participant flow from randomisation to primary analysis.

Altogether, fifty-six participants completed the full study protocol (Fig. 1). The dropouts did not differ from the completers apart from being more likely self-employed and of higher household asset score (online Supplementary Table 3). Of the dropouts, six (86 %) occurred during a Traditional dietary period, which for five (71 %) was the allocation for the first period (Fig. 1). The single dropout that occurred during a Western dietary intervention happened in the second intervention period.

### Diet

The dietary composition at baseline generally reflected a modern Greenlandic dietary pattern with Western influences, a high intake of protein and fat from traditional foods but also large amounts of imported meats and sugar-sweetened beverages (Table 2). Although the participants at all three sites consumed diets low in fruit and vegetables, Qaanaaq had especially low intake and their poorer diet was further characterised by a lower intake of fish and much more imported meat (online Supplementary Table 4).

**Table 2.** Dietary intake at baseline and during the dietary intervention period for 56 participants* (Medians and interquartile ranges; mean values and standard deviations)

	Baseline		Traditional		Western	
	Median	IQR	Median	IQR	Median	IQR
Total energy intake (kJ/d)	8042	2654	6790	2418	6853	2535
Protein (E%)						
Mean	20		24		18	
sd	4		8		4	
Carbohydrate (E%)						
Mean	51		45		52	
sd	9		14		11	
Fat (E%)						
Mean	29		30		30	
sd	6		9		8	
Total *n*-3 LCPUFA (g/d)	1·9	1·2, 3·7	4·6	2·4, 8·8	0·3	0·0, 0·9
Total marine mammal	32	11, 59	48	29, 103	5	0, 17
Seal	7	3, 20	13	3, 29	0	0, 3
Whale	4	1, 13	10	3, 29	0	0, 3
Muktuk	5	2, 15	15	7, 32	2	0, 7
Blubber	7	2, 10	10	3, 21	0	0, 2
Total fish	51	22, 85	91	48, 144	7	1, 14
Cod	7	1, 20	14	7, 34	0	0, 2
Halibut	10	3, 21	20	7, 39	0	0, 0
Trout/salmon	7	2, 11	14	7, 29	0	0, 0
Other fish	15	6, 27	29	15, 48	4	0, 7
Total traditional meat	15	6, 30	26	15, 48	1	0, 6
Reindeer/Musk	7	3, 20	17	7, 32	0	0, 3
Game bird	1	0, 3	0	0, 2	0	0, 0
Dried fish or meat	2	1, 5	4	2, 13	0	0, 1
Total imported meat	145	90, 215	38	13, 108	160	98, 258
Red meats	82	45, 172	25	9, 50	103	57, 172
Poultry	18	7, 29	3	0, 11	20	13, 43
Readymade meat meals	7	1, 14	0	0, 7	0	0, 14
Cold cuts	6	2, 14	3	1, 8	5	3, 9
Total cereal	86	57, 130	74	52, 114	86	56, 114
Rye bread	21	5, 101	11	0, 39	19	9, 46
Wheat bread	19	4, 58	1	0, 20	5	0, 22
Breakfast cereal and oats	19	6, 30	9	0, 23	3	0, 18
Pasta	13	2, 40	0	0, 16	0	0, 22
Rice	100	63, 166	52	22, 126	68	34, 150
Fruit and vegetable						
Apple/pear/banana	40	20, 70	33	11, 70	35	20, 70
Orange/grapefruit	32	9, 64	18	2, 39	32	14, 64
Other fruit	50	5, 142	35	0, 102	64	9, 124
Fruit Juice	29	12, 57	14	6, 43	29	13, 57
Vegetables	25	14, 43	18	9, 43	29	13, 43
Potato	229	177, 308	184	102, 259	233	127, 285
Dairy						
Dairy	99	7, 181	57	14, 150	107	20, 154
Cheese	25	15, 40	15	4, 40	24	11, 40
Ultra-processed foods (sweet)						
Cakes	5	2, 16	4	3, 8	8	4, 19
Sweets/chocolate	7	2, 21	4	1, 14	6	2, 15
Sweetened drinks	228	60, 415	119	29, 312	176	47, 333
Added sugars	12	0, 50	2	0, 28	0	0, 25
Ultra-processed foods (fatty)						
Fast food	9	3, 21	5	0, 18	6	0, 24
Crisps	5	1, 12	1	0, 5	1	0, 12

*n*-3 LCPUFA, long-chain *n*-3 fatty acid; E%: percentage of energy intake.

* Food intake in g/d (median and IQR) based on fifty-six participants with data from FFQ available for each visit, unrealistic diet entries (*n* 6) were removed. Estimated energy and nutrient intake presented as mean and sd.

The participants complied well with the interventions, supported by a 10-fold increase in consumption of traditional foods: marine mammal, fish and traditional meats during the Traditional *v*. the Western diet period. While consumption of imported meat was comparable between baseline and the Western dietary period, it was greatly reduced during the Traditional period. The difference in carbohydrate consumption was less pronounced between the intervention periods, but sugar intake was reduced in both periods compared with baseline. Estimated energy intake fell during both the intervention periods but E% from fat was comparable across baseline and the intervention visits (Table 2). Percentage energy from protein was greater, and from carbohydrate lower, in the Traditional *v*. the Western dietary period.

**Fig. 2.**
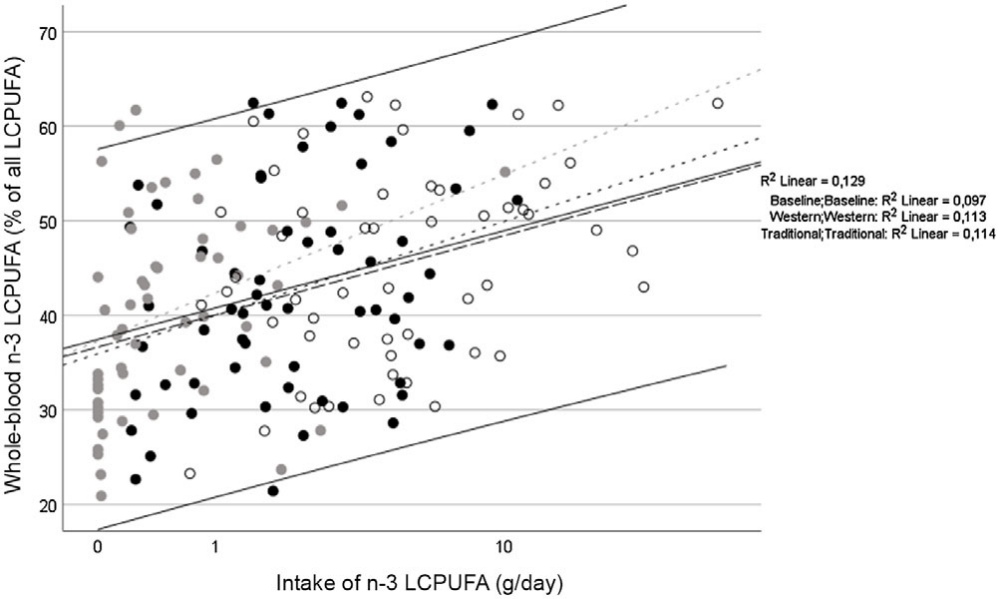
Correlation between the estimated intake of long-chain *n*-3 PUFA (*n*-3 LCPUFA) and whole-blood *n*-3 LCPUFA status. The solid line indicates the best fitted line across all periods with 95 % CI (*r* = 0·36, *P* < 0·001). The dotted lines are fit lines for the individual periods. Baseline: black dots and dotted line (*r* = 0·31); Western: gray dots and dotted line (*r* = 0·34) and Traditional: white dots and dashed line (*r* = 0·34).

The high compliance was verified by an agreement between FFQ and daily food logs, which was strong for fish and marine mammals (*r* = 0·51, *P* < 0·001) and imported foods (*r* = 0·42, *P* < 0·001), but not for cereal products (*r* = 0·11, *P* < 0·309). Although 22:6*n*-3 did not differ, 20:5*n*-3 did and the sum of the two increased from 3·0 (sd 1·5) % to 3·4 (sd 1·4) % of the fatty acids from the Western to the Traditional period, which verified compliance to the traditionally marine foods (online Supplementary Table 5). However, there was evidence (a high overall saturate to polyunsaturate ratio) that the samples were partially oxidised during handling and processing, most likely during storage at –20°C in Greenland. As a result, *n*-3 PUFA status and compliance were best reflected in the percentage of *n*-3 fatty acids among the LCPUFA (*n*-3 LCPUFA %), which was 4·6 (95 % CI 3·0; 6·2) %-point higher after the Traditional period (*P* < 0·001) and showed a correlation with the intake of traditional food during the past month (*r* = 0·28, *P* < 0·001) and the estimated intake of *n*-3 LCPUFA (Fig. 2).

### Glucose homoeostasis

We did not find any diet-induced change in 2-h post-OGTT plasma glucose, nor did we find differences between diet periods in the other measures from the OGTT at the population level (Table 3), but a genotype interaction was indicated for ISI_0,120_ (Table 4). At baseline, 2-h glucose was twice as high in carriers and their ISI_0,120_ values were about half of that in non-carriers, as expected, but there were no differences in any of the other indexes of insulin sensitivity, serum insulin or any of the fasting measures. These differences remained after the Traditional diet period but were much reduced after the Western period (Table 4), indicating a lower insulin sensitivity following the Traditional diet among carriers, but not non-carriers. No main effect of diet or genotype–interactions was observed for the other insulin sensitivity indexes: HOMA-IR, BIGTT-S_i_ and BIGTT-AIR, or 2-h insulin, fasting insulin and fasting glucose. In the whole population, HbA1c measured 5·5 % (sd 0·3), 5·6 % (sd 0·3) and 5·5 % (sd 0·3) at baseline and after the Traditional and Western period, respectively. Dietary period was not associated with a change in HbA1c (*β* = 0·04, (95 % CI −0·01, 0·09), *P* = 0·136) and there was no interaction observed with genotype (*P* = 0·368).

**Table 3.** Glucose homoeostasis at baseline and after the diet intervention periods and the estimated change between the interventions periods* (Medians and interquartile range; 95 % confidence intervals)

		Baseline		Traditional diet		Western diet		Difference†	95 % CI	*P*
	*n*	Median	IQR	Median	IQR	Median	IQR			
Fasting										
Glucose_0_ (mmol/l)	56	5·8	0·6, 6·1	5·7	0·6, 6·0	5·8	0·9, 6·2	–0·11	–0·27, 0·04	0·159
Insulin_0_ (pmol/l)	56	54	32, 105	61	37, 105	62	40, 115	–5	–17, 8	0·460
HOMA-IR	56	2·1	1·1, 3·7	2·1	1·3, 3·6	2·3	1·4, 4·4	–0·21	–0·69, 0·27	0·392
Oral glucose tolerance test										
Glucose_120_ (mmol/l)	56	5·8	4·8, 7·2	6·1	4·8, 7·9	6·1	4·7, 7·6	0·3	–0·1, 0·7	0·177
Insulin_120_ (pmol/l)	55	324	179, 506	291	180, 538	297	187, 512	–6	–76, 65	0·873
ISI_0,120_	55	1·6	1·3, 2·2	1·7	1·2, 2·1	1·7	1·3, 2·1	–0·01	–0·13, 0·12	0·925
BIGTT-S_I_	55	5·3	2·5, 8·0	5·3	3·0, 8·4	5·0	3·0, 7·8	0·21	–0·49, 0·92	0·552
BIGTT-AIR (×10^3^)	55	1·7	1·2, 2·6	1·9	1·4, 2·3	1·8	1·1, 3·0	–0·06	–0·29, 0·17	0·632
				Mean	sd	Mean	sd			
Continuous glucose monitor (mmol/l)	48									
Mean	48	–		5·2	0·5	5·4	0·6	–0·17	–0·29, −0·05	0·006
Max	48	–		8·2	1·1	8·5	1·0	–0·26	–0·46, −0·06	0·010
Min	48	–		3·7	0·5	3·8	0·6	–0·10	–0·23, 0·03	0·134
Range	48	–		4·5	1·0	4·7	1·0	–0·16	–0·34, 0·02	0·083

BIGTT, *β*-cell function insulin sensitivity glucose tolerance test (–AIR, acute insulin response and –S_i_, sensitivity index); HOMA-IR, insulin resistance assessed by the homoeostasis model; ISI_0.120_, insulin sensitivity index.

* Data are presented as mean and sd or median with IQR and estimated differences between diet intervention periods with 95 % CI.

† Differences between dietary interventions (Traditional – Western) estimated with linear mixed models. Models contain visit, order, baseline value (not available for continuous glucose monitor data) and random effects (site and individual). Non-normally distributed variables log-transformed (natural) prior to analysis and back-transformed using scaled back-transformation^(29)^.

**Table 4.** Glucose homoeostasis measures at baseline and after the diet periods stratified by genotype and estimated genotype-specific changes† (Medians and interquartile ranges; 95 % confidence intervals; mean values and standard deviations)

	Baseline					Traditional diet			
	Carriers		Non-carriers		*P*§	Carriers		Non-carriers	
Fasting	Median	IQR	Median	IQR		Median	IQR	Median	IQR
Glucose_0_ (mmol/l)	5·7	1·3, 6·3	5·9	0·5, 6·1	0·122	5·8	1·1, 5·4	5·7	0·4, 6·0
Insulin_0_ (pmol/l)	42	28, 86	65	33, 106	0·300	45	14, 53	66	40, 120
HOMA-IR	1·4	0·8, 3·3	2·2	1·3, 3·8	0·246	1·4	0·5, 2·6	2·2	1·4, 4·3
OGTT									
Glucose_120_ (mmol/l)	12·2	6·6, 13·9	5·5	4·8, 6·6	0·007	10·7	10·1, 13·9	5·6	4·7, 6·6
Insulin_120_ (pmol/l)	406	231, 863	300	179, 473	0·427	564	385, 672	270	175, 413
ISI_0,120_	0·9	0·8, 1·9	1·6	1·4, 2·2	0·036	1·0	1·0, 1·2	1·8	1·5, 2·3
BIGTT-S_I_	2·7	1·3, 9·3	5·4	3·0, 7·8	0·467	3·7	2·2, 5·7	5·4	3·6, 8·6
BIGTT-AIR||	1·7	1·1, 3·4	1·7	1·3, 2·5	0·891	1·6	1·4, 2·1	19·9	1·3, 2·3
CGM (mmol/l)						Mean	sd	Mean	sd
Mean	–		–		–	5·47	0·58	5·15	0·52
Max	–		–		–	9·54	0·75	8·02	0·93
Low	–		–		–	3·54	0·42	3·72	0·48
Range	–		–		–	5·97	0·63	4·30	0·86
	Western diet								
	Carriers		Non-carriers		Carrier		Non-carrier		Genotype interaction
Fasting	Median	IQR	Median	IQR	Difference‡	95 % CI	Difference‡	95 % CI	*P*
Glucose_0_ (mmol/l)	5·9	1·9, 5·4	5·8	0·5, 6·2	–0·22	–0·95, 0·52	–0·10	–0·22, 0·03	0·705
Insulin_0_ (pmol/l)	42	21, 75	65	44, 120	–12	–35, 11	–2	–16, 11	0·377
HOMA-IR	1·3	1·1, 2·5	2·3	1·5, 4·6	–0·46	–1·31, 0·39	–0·13	–0·63, 0·38	0·415
OGTT									
Glucose_120_ (mmol/l)	9·0	7·7, 11·4	5·4	4·7, 7·0	1·44	–0·63, 3·50	0·15	–0·24, 0·54	0·164
Insulin_120_ (pmol/l)	474	326, 548	267	170, 494	110	–48, 269	–17	–90, 55	0·338
ISI_0,120_	1·3	1·0, 1·5	1·8	1·4, 2·1	–0·15	–0·41, 0·11	0·04	–0·09, 0·17	0·093
BIGTT-S_I_	4·7	2·9, 5·3	5·2	3·0, 8·2	–0·12	–1·65, 1·42	0·34	–0·40, 1·08	0·483
BIGTT-AIR||	1·9	1·0, 2·8	1·7	1·1, 3·1	0·28	–0·30, 0·85	–0·13	–0·38, 0·12	0·188
CGM (mmol/l)	Mean	sd	Mean	sd					
Mean	5·63	0·85	5·34	0·51	–0·09	–0·37, 0·19	–0·18*	–0·30, −0·05	0·846
Max	9·70	1·24	8·33	0·84	–0·09	–0·38, 0·12	–0·29*	–0·51, −0·06	0·569
Low	3·71	0·82	3·82	0·51	–0·12	–0·55, 0·32	–0·10	–0·23, 0·04	0·741
Range	5·99	1·34	4·50	0·70	0·00	–0·65, 0·65	–0·18**	–0·37, 0·00	0·462

BIGTT, *β*-cell function insulin sensitivity glucose tolerance test (–AIR, acute insulin response and –S_i_, sensitivity index); CGM, continuous glucose monitor; HOMA-IR, insulin resistance assessed by the homoeostasis model; ISI_0.120_, insulin sensitivity index; OGTT, oral glucose tolerance test.

* *P* < 0·05.

** *P* = 0·05.

† Data are presented as mean and sd or median and IQR as well as estimated differences between diet intervention periods with 95 % confidence intervals based on *n* 9 carriers and *n* 47 non-carriers, except insulin_120_, ISI_0,120_ and the BIGTT indexes that had *n* 7 and *n* 46, respectively. CGM measures based on seven carriers and forty-two non-carriers.

‡ Differences between dietary interventions (Traditional – Western) estimated with linear mixed models. Models contain visit, order, baseline value (where available) and random effects (site and individual). Genotype interaction model additionally contains Gene × Intervention term. Non-normally distributed variables log-transformed (natural) prior to analysis and back-transformed using scaled back-transformation^(29)^.

§ Baseline differences between carriers and non-carriers tested by Student’s t and Kruskal–Wallis for normally and non-normally distributed variables.

|| BIGTT-AIR presented as ×10^–3^.

Despite similar energy intake in the two diet periods, participants lost an average of 0·5 kg body weight (*P* = 0·016) and 0·4 % body fat (*P* = 0·017) during the Traditional period relative to the Western period (Table 5). Adjusting the effect on ISI_0,120_ for the weight change slightly strengthened the genotype interaction (*P*_interaction_ *=* 0·078), which could be driven by a difference in Traditional diet-induced change in 2-h glucose clearance among carriers *v*. non-carriers, which is represented by ISI_0,120_ (online Supplementary Fig. 1).

**Table 5.** Cardio-metabolic markers at baseline and after Traditional and Western diet intervention periods* (95 % confidence intervals; mean values and standard deviations)

	*n*	Baseline		Traditional diet		Western diet		Difference†	95 % CI	*P*
		Mean	sd	Mean	sd	Mean	sd			
Total cholesterol (mmol/l)	56	5·81	1·05	5·74	1·10	5·75	1·15	0·02	–0·25, 0·21	0·870
LDL-cholesterol (mmol/l)	57	3·83	0·98	3·79	1·01	3·93	1·04	–0·14	–0·31, 0·04	0·121
HDL-cholesterol (mmol/l)	56	1·63	0·36	1·68	0·43	1·63	0·39	0·05	–0·01, 0·12	0·116
LDL:HDL	56	2·49	0·91	2·42	0·91	2·55	0·94	–0·13	–0·24, −0·04	0·018
TAG (mmol/l)										
Median	57	1·3		1·1		1·2		0·02	–0·08, 0·12	0·691
IQR		0·9, 1·6		0·9, 1·5		0·9, 1·4				
Systolic blood pressure (mmHg)	56	121	17	119	17	118	14	1·26	–2·65, 5·18	0·527
Diastolic blood pressure (mmHg)	56	75	9	75	9	74	10	1·66	–0·60, 3·92	0·152
CRP (mg/l)‡										
Median	49	1·3		1·2		1·1		0·11	–0·22, 0·45	0·509
IQR		0·5, 3·1		0·5, 2·0		0·4, 2·1				
Waist circumference (cm)	56	99·5	13·7	98·3	13·5	97·6	12·8	0·76	–0·36, 1·88	0·184
Weight (kg)	56	73·3	15·8	72·3	15·4	72·8	15·5	–0·49	–0·90, −0·09	0·016
Body fat (%)	56	31·7	10·3	31·0	10·1	31·4	10·1	–0·42	–0·76, −0·08	0·017

CRP: C-reactive protein.

* Data are presented as mean and sd or median (IQR) and estimated differences between diet intervention periods with (95 % confidence intervals).

† Differences between dietary interventions (Traditional – Western) estimated with linear mixed models. Models contain visit, order, baseline value, relevant medication (lipid meds for cholesterol and TAG models, hypertension meds for blood pressure models) and random effects (site and individual). Non-normally distributed variables log-transformed (natural) prior to analysis and back-transformed using scaled back-transformation^(29)^.

‡ Participants with acute infection, identified by CRP > 10 mg/l, at any visit removed from analysis.

**Fig. 3.**
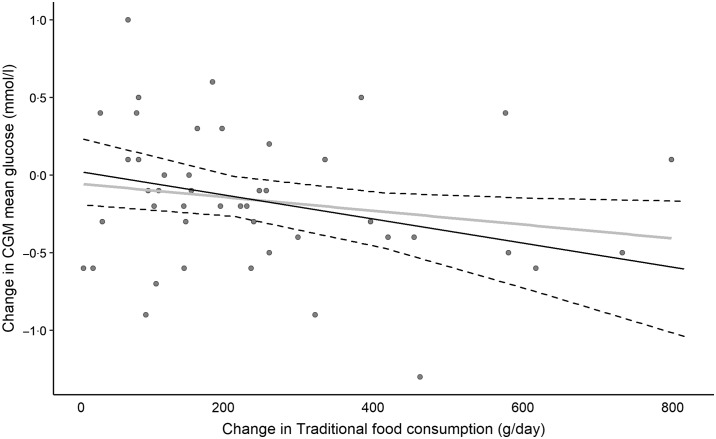
Correlation of change in traditional foods (from Western to traditional period) and change in continuous glucose monitor (CGM) mean glucose reading. Points indicate individual data (*n* 42, unrealistic dietary records removed), grey line represents unadjusted linear regression, black line represents adjusted regression with baseline weight, allocation order and period as covariates, dotted lines represent 95 % confidence limits. Adjusted regression indicates a 100 g increase in traditional foods corresponds to a 0·08 mmol/l reduction in CGM mean glucose (95 % CI −0·15, −0·00, *P* = 0·038).

In the overall population, we did, however, find a significant change in mean and maximum glucose readings during the first 14 d of the diet periods, which were both lower during the Traditional diet *v*. the Western diet period (Table 3). We found no genotype interactions for any of the outcomes from the CGM (Table 4). Daily minimum glucose readings were unaffected by diet and, accordingly, the daily glucose range for the population as a whole was lower in the Traditional diet period. A dose–response relationship was observed when change in CGM mean glucose was plotted against the change in Traditional food consumption between the two diets (Fig. 3). Adjusting for weight change or acute infection did not change the results for CGM outcomes.

### Cardio-metabolic risk markers

We found no diet-induced changes in BP, CRP, TAG, total cholesterol or on the absolute levels of LDL- and HDL-cholesterol (Table 5). However, the ratio of LDL to HDL was greater following the Western dietary period, which appeared to be driven by a non-significant decrease in HDL. The effect of the Traditional diet on HDL-cholesterol became slightly stronger when adjusted for acute infection (0·06 mmol/l (95 % CI 0·00, 0·13; *P* = 0·066), and the LDL:HDL-lowering effect remained at −0·11 (95 % CI −0·22, 0·00; *P* = 0·046). However, after adjusting for weight change the Traditional diet increased HDL-cholesterol by 0·09 mmol/l (95 % CI 0·03, 0·15; *P* = 0·006).

## Discussion

In this crossover RCT among the Greenland Inuit population, 2-h plasma glucose post oral glucose challenge was not different following a Traditional *v*. Westernised diet. However, daily glycaemic control was improved following a Traditional diet with reduced mean and max of blood glucose monitored over 14 d in a dose-dependent manner. In addition, the Traditional diet gave rise to weight loss and increased HDL. However, a potential gene–diet interaction was indicated on plasma glucose clearance in an OGTT, which contrary to our hypothesis could be reduced by a Traditional diet compared with a Westernised diet in homozygous carriers of the *TBC1D4* p.Arg684Ter variant.

The observed improvement in 14-d blood glucose is in line with a reported association between plasma *n*-3 LCPUFA and improved glucose tolerance in Native Alaskans, who were advised a traditional diet^(30)^. Studies in Alaskan Natives have also reported associations between daily consumption of seal oil and salmon and a lower IGT prevalence^(31)^, and between plasma *n*-3 LCPUFA and insulin sensitivity^(32)^. Furthermore, erythrocyte *n*-3 LCPUFA were inversely associated with HOMA-IR Greenland Inuit^(33)^. However, an 8-week RCT in healthy Danish men reported a doubling of erythrocyte LCPUFA but no change in HOMA-IR after 150 g/d trout compared with chicken controls^(34)^.

We provided a variety of foods and dietary advice, so it is difficult to extract which specific component(s) might have contributed to the results, but the high *n*-3 LCPUFA intake would be an obvious candidate. The observed increase in HDL after the Traditional diet is supported by RCT with fish^(35)^. TAG on the other hand was unaffected by diet, which is surprising considering the established TAG-lowering effects of fish oil^(36,37)^, and we found no benefits on BP, although this effect is well-supported by a large meta-analysis of RCT with fish and fish oil^(38)^. The observed weight loss could contribute to increased insulin sensitivity^(39)^, however, control for weight change did not alter the effects on glycaemic control.

Meta-analyses of RCT with fish oil supplementation have not shown any effect on measures of glucose homoeostasis^(40,41)^, but insulin sensitisation and even reversal of IGT by *n*-3 LCPUFA are well-supported in murine models^(42,43)^. Proposed mechanisms include intramuscular lipid clearance^(44,45)^ and modulation of gene expression in the liver and skeletal muscle^(46)^. Intake of *n*-3 LCPUFA is known to up-regulate thermogenic *Ucp1* expression and induce adipocyte browning in rodents^(47)^. The *n*-3 LCPUFA are the natural ligands of PPAR-*α* and -γ^(48)^, which regulates gene transcription that affects the metabolism of both lipid and glucose^(49)^. PPAR-γ has been shown to affect transcription of components in the insulin-signalling cascade^(50)^ and one could imagine it might also affect compensatory mechanisms of GLUT4 translocation relied on by p.Arg684Ter variant homozygotes. None of the previous studies has considered the influence of *TBC1D4* genotype, but the suggested decrease in ISI_0,120_ and 2-h glucose clearance after the traditional diet indicates a potential decrease in insulin sensitivity in already impaired skeletal muscle of the carriers^(24)^. An RCT by Mostad *et al.* showed a fish oil-induced elevation in plasma glucose that could be explained by a shift in oxidation from carbohydrate to fat^(51)^. We suspect that an adaption to low carbohydrate during the Traditional diet period could be detrimental in handling the sugar bolus of the OGTT, specifically in carriers of the p.Arg684Ter variant, thus reducing glucose clearance. On the other hand, CGM measures glycaemic fluctuations depending on the everyday diet, which could give rise to contrasting results in the CGM and OGTT measures. While the tests performed on CGM and OGTT data used different sample sizes due to missing data, the significant findings within the smaller sample size (CGM) suggest that this is not the cause of contrasting outcomes.

The glycaemic index of the Traditional diet is likely lower than in the Western diet given the increased E% from protein, as well as reduced intake of sweetened beverages, which could reduce daily fluctuations in peak glucose. The main difference between the two periods was an exchange of imported meat with marine sources, but total E% from protein was higher in the Traditional diet. Beneficial effects of isolated fish protein on glycaemic control^(52,53)^ and markers of IGT^(54)^ have been reported in RCT.

The provided foods covered > 20 % of the participant’s daily energy intake which, together with a manageable intervention period, promoted low dropout and high compliance. The whole-blood fatty acid analyses verify the increase in *n*-3 LCPUFA intake during the Traditional diet period, although the differences in the markers of intake were small. These objective markers of compliance help to support the self-reported dietary intake data which are unavoidably subjective in nature. The *n*-3 LCPUFA intake at baseline was high, which limited the establishment of compliance with whole-blood *n*-3 LCPUFA and it may also have limited the detection of effects of the increased intake from the Traditional diet. Furthermore, we compare to a relatively healthy Western diet, similar to that of Denmark, which included rye bread, oatmeal and lean meats rather than often-associated processed foods. The 4-week duration of the intervention periods was sufficiently long to see changes in FA biomarkers and would generally be considered long enough to detect metabolic effects of changes in dietary macronutrient composition, so extending the durations may not necessarily have resulted in greater magnitudes of difference. The crossover design and complete-case analysis minimised the influence of between-subject variability and potential bias due to dropout, which increase the power of the study. We did not adjust for multiple testing, but the primary outcome was pre-specified and additional measures of glycaemic control were included to examine consistency and mechanisms, so a multiplicity adjustment would have been overly conservative. The trial was not powered to examine interactions, but the stratified analyses were pre-planned. Poor recruitment among carriers resulted underpowered analysis within that group specifically and so the results we present should be interpreted with caution. Effect sizes and confidence intervals may therefore be more relevant than *P*-values. We aimed to be able to detect a difference of −1·1 mmol/l in 2-h glucose within thirty carriers and observed non-significant difference of +1·4 mmol/l in only seven, so the lack of significance could be ascribed to a type 2 error. Diabetes based on an assessment of HbA1c was an exclusion criterion, but the carriers had greatly elevated glucose 2-h post-OGTT to an extent that indicated that 60 % had IGT or even diabetes, which is in line with previous findings^(5)^. Including participants far into the progression of IGT may impair the relevance of the results in terms of T2D prevention. Furthermore, the accuracy of the BIGTT indexes is known to weaken in people with diabetes^(25)^, possibly undermining the application of this measure in the participants. The exclusion of subjects with T2D has on the other hand resulted in a selective inclusion of an unrepresentative healthier sample, especially of the carriers as they have an increased risk of T2D.

A major strength of the study is the real-life *ad libitum* diet intervention carried out at three sites in different seasons, thus encompassing diet variation and ensuring participant heterogeneity. The *n*-3 LCPUFA intake in the Traditional diet was around 4·6 g/d, which is similar to the reported intake in Greenland Inuit in 1976^(55)^ and baseline diets were typical of modern Inuit^(22)^, so the observed effects could likely be generalised to the population. Mean daily glucose levels improved on the Traditional diet, although more so among the larger sample of non-carriers, we suspect this observation may be due to low power in the carriers analysis. The short study duration precludes inference of the results for long-term prevention, but it is possible that the Traditional diet can reduce T2D development due to effects on body weight and lipid metabolism. Furthermore, reduced blood glucose variation may confer protection from vascular complications associated with T2D^(56)^.

In conclusion, the Traditional Inuit diet improved daily glycaemic control compared with a Western diet, which indicates that it may slow the progression of glucose intolerance over time and may represent a tool for counteracting the ongoing diabetes epidemic in Greenland. However, our results suggest that homozygous carriers of the *TBC1D4* p.Arg684Ter variant might experience reductions in glucose tolerance in OGTT, which warrants further studies on potential diet–gene interactions in the development of T2D.

## Supplementary material

For supplementary material accompanying this paper visit https://doi.org/10.1017/S000711452300106X. click here to view supplementary material
