# Supplementary material for: The effect of traditional diet on glucose homoeostasis in carriers and non-carriers of a common TBC1D4 variant in Greenlandic Inuit: a randomised crossover study
Source: Br J Nutr. 2023 May 2;130(11):1871–84. doi: 10.1017/S000711452300106X (PMC10632723; doi:10.1017/S000711452300106X)
Supplement: Supplementary file 1 [file S000711452300106Xsup001.docx]

| **Supplementary Table 1.** Baseline characteristics of participants randomized to receive either Traditional or Western diets first^a^ | | | |
| --- | --- | --- | --- |
|  | **Traditional**  (*n* = 33) | **Western**  (*n* = 30) | *P*^b^ |
| Homozygous carriers | 6 (18.2%) | 5 (16.7%) | 0.874 |
| Female | 18 (54.5%) | 16 (53.3%) | 0.923 |
| Age (years) | 57 ± 11 | 56 ± 9 | 0.790 |
| Anthropometrics |  |  |  |
| Height (cm) | 163 ± 10 | 165 ± 9 | 0.493 |
| Weight (kg) | 75 ± 20 | 75 ± 14 | 0.955 |
| BMI (kg/m^2^) | 27.8 ± 6.2 | 27.4 ± 4.6 | 0.803 |
| FFM (kg) | 50.4 ± 12.1 | 50.0 ± 9.3 | 0.878 |
| Body fat (%) | 31.1 ± 10.5 | 32.2 ± 9.8 | 0.665 |
| Waist circumference (cm) | 99 ± 16 | 101 ± 12 | 0.701 |
| Hip circumference (cm) | 103 ± 10 | 104 ± 9 | 0.593 |
| Cardio-metabolic risk factors |  |  |  |
| Systolic blood pressure (mmHg) | 122 ± 17 | 119 ± 16 | 0.535 |
| Diastolic blood pressure (mmHg) | 75 ± 9 | 74 ± 9 | 0.634 |
| Pulse (bpm) | 75 ± 11 | 73 ± 11 | 0.460 |
| Total cholesterol (mmol/L) | 6.0 ± 1.1 | 5.8 ± 1.1 | 0.445 |
| HDL-cholesterol (mmol/L) | 1.70 ± 0.40 | 1.53 ± 0.28 | 0.061 |
| LDL-cholesterol (mmol/L) | 3.94 ± 0.99 | 3.89 ± 1.02 | 0.828 |
| TG (mmol/L) | 1.3 (1.1, 1.6) | 1.2 (0.9, 1.6) | 0.880 |
| CRP (mg/L) | 1.3 (0.5, 2.5) | 1.7 (0.7, 3.3) | 0.265 |
| HbA1c (%) | 5.6 (5.3, 5.8) | 5.5 (5.4, 5.7) | 0.945 |
| Previous diagnosis |  |  |  |
| Hypertension | 10 (30.3%) | 16 (57.1%) | 0.035 |
| Heart clot | 3 (9.1%) | 1 (3.3%) | 0.349 |
| Heart disease | 5 (15.6%) | 4 (13.3%) | 0.798 |
| Brain clot | 6 (18.2%) | 0 (0.0%) | 0.014 |
| Asthma | 6 (18.2%) | 0 (0.0%) | 0.014 |
| COPD | 4 (12.5%) | 0 (0.0%) | 0.045 |
| Recent antibiotics | 3 (10.7%) | 4 (14.3%) | 0.686 |
| Smoking status |  |  |  |
| Current smoker | 19 (57.6%) | 18 (60.0%) |  |
| Previous smoker | 13 (39.4%) | 7 (23.3%) |  |
| Never smoked | 1 (3.0%) | 5 (16.7%) | 0.113 |
| Alcohol frequency |  |  |  |
| ≤ once per month | 20 (62.5%) | 18 (60.0%) |  |
| 2-4 times per month | 9 (28.1%) | 10 (33.3%) |  |
| ≥ 2 times per week | 3 (9.4%) | 2 (6.7%) | 0.863 |
| Physical activity (MET min x10^3^) | 5.2 (2.7, 7.8) | 4.6 (3.3, 10.9) | 0.596 |
| Employment type |  |  |  |
| Full time | 19 (57.6%) | 20 (66.7%) |  |
| Self-employed | 3 (9.1%) | 1 (3.3%) |  |
| Pensioner | 7 (21.2%) | 4 (13.3%) |  |
| Other | 4 (12.1%) | 5 (16.7%) | 0.611 |
| Asset score | 7 ± 2 | 7 ± 2 | 0.713 |
| ^a^ Data are given as mean ± SD for normally distributed continuous variables, median (IQR) for non-normally distributed continuous variables and number (%) for categorical variables.  ^b^ Level of significance for differences between genotype groups assessed by linear model ANOVA and Kruskal-Wallis rank sum test for normally and non-normally distributed continuous variables, respectively, and by Pearson's Chi-squared test for categorical variables  ^c^ In the last 3 months.  Abbreviations: COPD: Chronic obstructive pulmonary disease; CRP: C-reactive protein, FFM: Fat free mass; HbA1c: glycated hemoglobin, MET: metabolic equivalents; TG: triglyceride | | | |

| **Supplementary Table 2.** Baseline characteristics of enrolled participants at each site^a^ | | | | | |
| --- | --- | --- | --- | --- | --- |
|  | **Nuuk**  *(n = 13)* | **Qaanaaq**  *(n = 19)* | **Qasigiannguit**  *(n = 31)* |  | *P*^b^ |
| Traditional – Western allocation | 6 (46%) | 12 (63%) | 15 (48%) |  | 0.526 |
| Homozygous carrier | 5 (39%) | 2 (11%) | 4 (13%) |  | 0.080 |
| Female | 7 (54%) | 9 (47%) | 18 (58%) |  | 0.762 |
| Age (years) | 55 ± 8 | 57 ± 8 | 57 ± 12 |  | 0.755 |
| Anthropometrics |  |  |  |  |  |
| Height (cm) | 166 ± 5 | 163 ± 10 | 164 ± 11 |  | 0.617 |
| Weight (kg) | 81 ± 16 | 72 ± 17 | 74 ± 18 |  | 0.311 |
| BMI (kg/m^2^) | 29.2 ± 5.1 | 27.1 ± 6.2 | 27.2 ± 5.1 |  | 0.471 |
| FFM (kg) | 52.0 ± 9.6 | 49.4 ± 10.3 | 50.0 ± 11.7 |  | 0.789 |
| Body fat (%) | 35.1 ± 8.0 | 29.8 ± 10.5 | 31.2 ± 10.5 |  | 0.323 |
| Waist circumference (cm) | 105 ± 12 | 96 ± 16 | 100 ± 13 |  | 0.217 |
| Hip circumference (cm) | 105 ± 8 | 101 ± 10 | 104 ± 9 |  | 0.416 |
| Cardio-metabolic risk factors |  |  |  |  |  |
| Systolic blood pressure (mmHg) | 115 ± 13 | 118 ± 18 | 126 ± 16 |  | 0.062 |
| Diastolic blood pressure (mmHg) | 71 ± 5 | 72 ± 11 | 78 ± 8 |  | 0.010 |
| Pulse (bpm) | 75 ± 13 | 76 ± 10 | 73 ± 11 |  | 0.618 |
| Total cholesterol (mmol/L) | 5.48 ± 1.01 | 6.22 ± 1.07 | 5.87 ± 1.05 |  | 0.152 |
| HDL-cholesterol (mmol/L) | 1.57 ± 0.29 | 1.84 ± 0.33 | 1.51 ± 0.34 |  | 0.004 |
| LDL-cholesterol (mmol/L) | 3.50 ± 0.82 | 4.16 ± 1.01 | 3.94 ± 1.03 |  | 0.182 |
| TG (mmol/L) | 1.3 (1.1, 1.5) | 1.4 (0.9, 1.5) | 1.3 (1.0, 1.7) |  | 0.810 |
| CRP (mg/L) | 1.3 (0.8, 2.5) | 0.7 (0.5, 3.3) | 1.8 (0.7, 3.0) |  | 0.674 |
| HbA1c (%) | 5.6 (5.4, 5.8) | 5.5 (5.4, 5.7) | 5.5 (5.4, 5.7) |  | 0.482 |
| Previous diagnosis |  |  |  |  |  |
| Hypertension | 3 (23.1%) | 10 (52.6%) | 13 (44.8%) |  | 0.239 |
| Heart clot | 2 (15.4%) | 1 (5.3%) | 1 (3.2%) |  | 0.312 |
| Heart disease | 2 (15.4%) | 2 (10.5%) | 5 (16.7%) |  | 0.834 |
| Brain clot | 2 (15.4%) | 2 (10.5%) | 2 (6.5%) |  | 0.644 |
| Asthma | 2 (15.4%) | 1 (5.3%) | 3 (9.7%) |  | 0.631 |
| COPD | 3 (23.1%) | 0 (0.0%) | 1 (3.3%) |  | 0.021 |
| Recent antibiotics^c^ | 0 (0.0%) | 5 (29.4%) | 2 (6.7%) |  | 0.036 |
| Smoking status |  |  |  |  |  |
| Current smoker | 7 (53.8%) | 13 (68.4%) | 17 (54.8%) |  |  |
| Previous smoker | 5 (38.5%) | 5 (26.3%) | 10 (32.3%) |  |  |
| Never smoked | 1 (7.7%) | 1 (5.3%) | 4 (12.9%) |  | 0.812 |
| Alcohol frequency |  |  |  |  |  |
| ≤ once per month | 10 (76.9%) | 11 (57.9%) | 17 (56.7%) |  |  |
| 2-4 times per month | 2 (15.4%) | 6 (31.6%) | 11 (36.7%) |  |  |
| ≥ 2 times per week | 1 (7.7%) | 2 (10.5%) | 2 (6.7%) |  | 0.695 |
| Physical activity (MET min x10^3^) | 3.4 (2.8, 6.2) | 4.0 (1.4, 6.1) | 8.1 (4.6, 11.5) |  | 0.012 |
| Employment type |  |  |  |  |  |
| Full time | 11 (84.6%) | 12 (63.2%) | 16 (51.6%) |  |  |
| Self-employed | 0 (0.0%) | 0 (0.0%) | 4 (12.9%) |  |  |
| Pensioner | 1 (7.7%) | 3 (15.8%) | 7 (22.6%) |  |  |
| Other | 1 (7.7%) | 4 (21.1%) | 4 (12.9%) |  | 0.238 |
| Asset score | 7 ± 2 | 6 ± 2 | 7 ± 2 |  | 0.161 |
| ^a^ Data are given as mean ± SD for normally distributed continuous variables, median (IQR) for non-normally distributed continuous variables and number (%) for categorical variables.  ^b^ Level of significance for differences between genotype groups assessed by linear model ANOVA and Kruskal-Wallis rank sum test for normally and non-normally distributed continuous variables, respectively, and by Pearson's Chi-squared test for categorical variables  ^c^ In the last 3 months.  Abbreviations: COPD: Chronic obstructive pulmonary disease; CRP: C-reactive protein, FFM: Fat-free mass; HbA1c: glycated hemoglobin, MET: metabolic equivalents; TG: triglyceride | | | | | |

| **Supplementary Table 3.** Baseline characteristics of dropouts and completers^a^ | | | |
| --- | --- | --- | --- |
|  | **Dropouts**  (*n* = 7) | **Completers**  (*n* = 56) | *P*^b^ |
| Traditional – Western Allocation | 6 (85.7%) | 27 (48.2%) | 0.061 |
| Homozygous carrier | 2 (28.6%) | 9 (16.1%) | 0.411 |
| Female | 3 (42.9%) | 31 (55.4%) | 0.532 |
| Age (years) | 53 ± 8 | 57 ± 10 | 0.295 |
| Anthropometrics |  |  |  |
| Height (cm) | 170 ± 12 | 163 ± 9 | 0.075 |
| Weight (kg) | 85 ± 26 | 73 ± 16 | 0.095 |
| BMI (kg/m^2^) | 29.1 ± 6.7 | 27.4 ± 5.3 | 0.445 |
| FFM (kg) | 57.9 ± 17.0 | 49.3 ± 9.5 | 0.046 |
| Body fat (%) | 31.3 ± 9.1 | 31.7 ± 10.3 | 0.929 |
| Waist circumference (cm) | 103 ± 16 | 100 ± 14 | 0.488 |
| Hip circumference (cm) | 105 ± 8 | 103 ± 9 | 0.490 |
| Cardio-metabolic risk factors |  |  |  |
| Systolic blood pressure (mmHg) | 121 ± 12 | 121 ± 17 | 0.974 |
| Diastolic blood pressure (mmHg) | 76 ± 7 | 75 ± 9 | 0.644 |
| Pulse (bpm) | 75 ± 14 | 74 ± 11 | 0.821 |
| Total cholesterol (mmol/L) | 6.61 ± 1.03 | 5.81 ± 1.05 | 0.060 |
| HDL-cholesterol (mmol/L) | 1.56 ± 0.35 | 1.63 ± 0.36 | 0.653 |
| LDL-cholesterol (mmol/L) | 4.59 ± 0.88 | 3.83 ± 0.99 | 0.057 |
| TG (mmol/L) | 1.2 (1.0, 1.6) | 1.3 (0.9, 1.6) | 0.930 |
| CRP (mg/L) | 1.4 (0.6, 2.7) | 1.4 (0.5, 2.9) | 0.870 |
| HbA1c (%) | 5.6 (5.5, 5.9) | 5.5 (5.4, 5.7) | 0.295 |
| Previous diagnosis |  |  |  |
| Hypertension | 1 (14.3%) | 25 (46.3%) | 0.107 |
| Heart clot | 0 (0.0%) | 4 (7.1%) | 0.465 |
| Heart disease | 0 (0.0%) | 9 (16.4%) | 0.247 |
| Brain clot | 0 (0.0%) | 6 (10.7%) | 0.363 |
| Asthma | 2 (28.6%) | 4 (7.1%) | 0.069 |
| COPD | 0 (0.0%) | 4 (7.3%) | 0.461 |
| Recent antibiotics | 0 (0.0%) | 7 (13.5%) | 0.433 |
| Smoking status |  |  |  |
| Current smoker | 4 (57.1%) | 33 (58.9%) |  |
| Previous smoker | 3 (42.9%) | 17 (30.4%) |  |
| Never smoked | 0 (0.0%) | 6 (10.7%) | 0.589 |
| Alcohol frequency |  |  |  |
| ≤ once per month | 3 (50.0%) | 35 (62.5%) |  |
| 2-4 times per month | 3 (50.0%) | 16 (28.6%) |  |
| ≥ 2 times per week | 0 (0.0%) | 5 (8.9%) | 0.476 |
| Physical activity (MET min x10^3^) | 4.0 (2.9, 4.9) | 5.6 (3.2, 9.2) | 0.450 |
| Employment type |  |  |  |
| Full time | 4 (57.1%) | 35 (62.5%) |  |
| Self-employed | 3 (42.9%) | 1 (1.8%) |  |
| Pensioner | 0 (0.0%) | 11 (19.6%) |  |
| Other | 0 (0.0%) | 9 (16.1%) | <0.001 |
| Asset score | 9 ± 1 | 7 ± 2 | 0.008 |
| ^a^ Data are given as mean ± SD for normally distributed continuous variables, median (IQR) for non-normally distributed continuous variables and number (%) for categorical variables.  ^b^ Level of significance for differences between genotype groups assessed by linear model ANOVA and Kruskal-Wallis rank sum test for normally and non-normally distributed continuous variables, respectively, and by Pearson's Chi-squared test for categorical variables  ^c^ In the last 3 months.  Abbreviations: COPD: Chronic obstructive pulmonary disease; CRP: C-reactive protein, FFM: Fat free mass; HbA1c: glycated hemoglobin, MET: metabolic equivalents; TG: triglyceride | | | |

| **Supplementary Table 4**. Baseline intake of typically occurring foods in the three study sites^a^ | | | | |
| --- | --- | --- | --- | --- |
|  | Nuuk | Qaanaaq | Qasigiannguit | All |
| Marine mammal |  |  |  |  |
| Seal | 4 [2, 7] | 7 [2, 18] | 10 [5, 29] | 7 [3, 20] |
| Whale | 3 [1, 7] | 7 [1, 13] | 7 [2, 13] | 7 [1, 13] |
| Mattak | 7 [2, 29] | 13 [3, 29] | 3 [1, 7] | 7 [2, 20] |
| Blubber | 7 [3, 14] | 2 [1, 7] | 7 [3, 14] | 7 [2, 14] |
| *Total marine mammal* | 27 [10, 50] | 35 [8, 78] | 40 [15, 61] | 35 [11, 59] |
| Fish |  |  |  |  |
| Cod | 13 [10, 20] | 0 [0, 1] | 10 [7, 20] | 7 [1, 20] |
| Halibut | 10 [3, 20] | 5 [2, 16] | 10 [7, 27] | 10 [3, 20] |
| Trout / salmon | 7 [3, 13] | 2 [1, 8] | 7 [2, 10] | 7 [2, 10] |
| Other fish | 20 [8, 36] | 4 [1, 13] | 24 [17, 36] | 17 [7, 28] |
| *Total fish* | 67 [40, 89] | 20 [10, 38] | 60 [47, 90] | 53 [24, 85] |
| Meat (traditional) |  |  |  |  |
| Reindeer / Musk | 10 [7, 20] | 3 [1, 7] | 10 [6, 27] | 7 [3, 20] |
| Game bird | 1 [1, 2] | 1 [1, 3] | 2 [0, 4] | 1 [0, 3] |
| Dried fish or meat | 4 [1, 4] | 1 [1, 3] | 3 [1, 6] | 2 [1, 5] |
| *Total meat (traditional)* | 16 [12, 29] | 5 [3, 19] | 21 [11, 42] | 15 [5, 28] |
| Meat (imported) |  |  |  |  |
| Red meats | 73 [57, 191] | 121 [70, 214] | 59 [43, 121] | 86 [46, 172] |
| Poultry | 14 [10, 29] | 26 [10, 36] | 14 [7, 29] | 20 [7, 29] |
| Readymade meat meals | 2 [0, 29] | 10 [3, 60] | 6 [0, 10] | 7 [1, 15] |
| Cold cuts | 9 [4, 20] | 6 [1, 11] | 8 [2, 20] | 6 [3, 14] |
| *Total meat (imported)* | 122 [104, 223] | 210 [152, 286] | 124 [71, 162] | 143 [90, 215] |
| Cereal and cereal product |  |  |  |  |
| Rye bread | 50 [35, 70] | 20 [10, 70] | 60 [35, 90] | 45 [20, 70] |
| Wheat bread | 41 [18, 64] | 37 [14, 64] | 32 [9, 64] | 32 [9, 64] |
| Breakfast cereal & oats | 64 [13, 107] | 8 [2, 69] | 82 [27, 158] | 51 [7, 134] |
| Pasta | 29 [13, 57] | 57 [14, 107] | 14 [7, 43] | 29 [9, 57] |
| Rice | 29 [14, 43] | 21 [5, 43] | 15 [14, 43] | 21 [14, 43] |
| *Total cereal* | 219 [183, 254] | 222 [132, 309] | 246 [199, 308] | 240 [178, 308] |
| Fruit and Vegetable |  |  |  |  |
| Apple / pear / banana | 39 [19, 116] | 10 [3, 20] | 44 [16, 135] | 21 [7, 106] |
| Orange / grapefruit | 39 [19, 75] | 10 [4, 19] | 27 [7, 110] | 19 [4, 66] |
| Other fruit | 22 [10, 39] | 3 [1, 17] | 22 [9, 46] | 19 [5, 37] |
| Fruit Juice | 29 [0, 29] | 6 [1, 43] | 13 [3, 29] | 13 [2, 40] |
| Vegetables | 139 [74, 221] | 69 [30, 134] | 113 [76, 186] | 105 [65, 169] |
| Potato | 86 [74, 114] | 74 [47, 104] | 93 [74, 143] | 86 [57, 130] |
| Dairy |  |  |  |  |
| Dairy | 21 [0, 150] | 64 [1, 157] | 150 [27, 230] | 150 [9, 230] |
| Cheese | 25 [17, 40] | 21 [9, 24] | 25 [15, 45] | 25 [15, 40] |
| Ultra-processed foods *(sweet)* |  |  |  |  |
| Cakes | 8 [4, 31] | 4 [2, 20] | 8 [4, 16] | 8 [2, 16] |
| Sweets / chocolate | 4 [2, 22] | 14 [3, 46] | 7 [2, 11] | 7 [2, 21] |
| Sweetened drinks | 202 [40, 357] | 236 [113, 482] | 216 [72, 374] | 228 [55, 409] |
| Added sugars | 0 [0, 30] | 40 [0, 60] | 10 [0, 20] | 15 [0, 50] |
| *(fatty)* |  |  |  |  |
| Fast food | 14 [5, 28] | 8 [4, 21] | 10 [3, 21] | 10 [3, 24] |
| Crisps | 1 [1, 6] | 11 [6, 17] | 3 [1, 6] | 6 [1, 11] |
| ^a^ Food intake in g/day based on the 63 enrolled participants with data from food frequency questionnaire available for each visit, unrealistic diet entries (6) removed. The estimated energy and nutrient intake is given as mean ± SD or median (IQR).  Abbreviations. n-3 LCPUFA: long-chain n-3 fatty acid; E%: percentage of energy intake. | | | | |

**Supplementary Table 5.** Whole-blood fatty acid composition at baseline and after each of the two dietary intervention period^a^

|  | **Baseline**  (*n = 63)* | **Traditional**  (*n = 57*) | **Western**  (*n = 57*) | *P*^b^ |
| --- | --- | --- | --- | --- |
| 14:0 | 0.73 ± 0.20 | 0.81 ± 0.25 | 0.72 ± 0.22 | 0.036 |
| 15:0 | 0.25 ± 0.06 | 0.28 ± 0.06 | 0.27 ± 0.07 | 0.186 |
| 16:0 | 23.97 ± 1.62 | 24.22 ± 1.95 | 24.07 ± 1.63 | 0.535 |
| 17:0 | 0.31 ± 0.07 | 0.32 ± 0.06 | 0.31 ± 0.07 | 0.713 |
| 18:0 | 12.13 ± 1.20 | 11.94 ± 1.39 | 12.29 ± 1.25 | 0.023 |
| 20:0 | 0.39 ± 0.06 | 0.37 ± 0.07 | 0.37 ± 0.06 | 0.882 |
| 22:0 | 1.18 ± 0.24 | 1.10 ± 0.29 | 1.18 ± 0.22 | 0.011 |
| 23:0 | 0.30 ± 0.08 | 0.31 ± 0.08 | 0.32 ± 0.08 | 0.364 |
| 24:0 | 2.08 ± 0.48 | 1.96 ± 0.58 | 2.11 ± 0.42 | 0.007 |
| SFA | 41.48 ± 2.57 | 41.48 ± 3.09 | 41.80 ± 2.48 | 0.366 |
| 16:1 | 1.96 ± 0.77 | 2.28 ± 0.98 | 1.91 ± 0.70 | 0.000 |
| 18:1n-7 | 1.70 ± 0.21 | 1.83 ± 0.28 | 1.76 ± 0.30 | 0.156 |
| 18:1n-9 | 20.40 ± 2.13 | 20.50 ± 2.02 | 20.54 ± 2.62 | 0.971 |
| 20:1n-9 | 0.44 ± 0.15 | 0.55 ± 0.22 | 0.46 ± 0.25 | 0.021 |
| 22:1n-9 | 0.18 ± 0.06 | 0.22 ± 0.09 | 0.20 ± 0.07 | 0.141 |
| 24:1n-9 | 3.24 ± 0.58 | 3.29 ± 0.68 | 3.32 ± 0.64 | 0.583 |
| MUFA | 27.98 ± 2.48 | 28.75 ± 2.38 | 28.27 ± 2.75 | 0.155 |
| 18:2n-6 | 12.81 ± 2.09 | 12.01 ± 2.11 | 12.39 ± 2.17 | 0.198 |
| 18:3n-6 | 0.11 ± 0.04 | 0.10 ± 0.05 | 0.11 ± 0.05 | 0.230 |
| 20:2n-6 | 0.20 ± 0.04 | 0.19 ± 0.04 | 0.21 ± 0.05 | 0.007 |
| 20:3n-6 | 1.02 ± 0.28 | 0.86 ± 0.29 | 0.99 ± 0.27 | 0.001 |
| 20:4n-6 | 3.81 ± 0.79 | 3.52 ± 0.70 | 3.76 ± 1.23 | 0.099 |
| 22:4n-6 | 0.15 ± 0.06 | 0.16 ± 0.06 | 0.16 ± 0.07 | 0.981 |
| 22:5n-6 | 0.36 ± 0.14 | 0.36 ± 0.15 | 0.41 ± 0.18 | 0.041 |
| *n*-6 PUFA | 0.13 ± 0.06 | 0.13 ± 0.05 | 0.14 ± 0.06 | 0.246 |
| 18:3n-3 | 0.25 ± 0.07 | 0.25 ± 0.08 | 0.24 ± 0.07 | 0.000 |
| 20:5n-3 | 0.98 ± 0.75 | 1.09 ± 0.77 | 0.83 ± 0.66 | 0.007 |
| 22:5n-3 | 0.83 ± 0.22 | 0.84 ± 0.25 | 0.81 ± 0.27 | 0.433 |
| 22:6n-3 | 2.41 ± 0.87 | 2.32 ± 0.75 | 2.16 ± 0.87 | 0.148 |
| *n*-3 PUFA | 4.52 ± 1.72 | 4.54 ± 1.68 | 4.10 ± 1.74 | 0.033 |
| 20:3n-9 | 0.10 ± 0.03 | 0.09 ± 0.03 | 0.10 ± 0.04 | 0.015 |
| PUFA | 23.20 ± 3.18 | 21.96 ± 3.27 | 22.37 ± 3.74 | 0.385 |
| LCPUFA | 9.69 ± 1.98 | 9.26 ± 1.93 | 9.27 ± 2.63 | 0.930 |
| EPA + DHA | 3.39 ± 1.52 | 3.40 ± 1.45 | 3.00 ± 1.49 | 0.015 |
| *n-*6:*n-*3 PUFA | 4.75 ± 1.92 | 4.39 ± 1.89 | 5.15 ± 2.02 | 0.001 |
| n-3 LCPUFA % | 43 ± 11 | 45 ± 11 | 41 ± 10 | 0.000 |
| Identification (%) | 93 ± 2 | 92 ± 2 | 92 ± 2 | 0.411 |

^a^ Data are given as percentage of all fatty acids (mean ± SD)

^b^Paired t-tests comparing Traditional and Western periods

Abbreviations: SFA: saturated fatty acids, MUFA: monounsaturated fatty acids, PUFA: polyunsaturated fatty acids, LCPUFA: long-chain PUFA (≥20 carbons, ≥3 double bonds), n-3 LCPUFA %: n-3 LCPUFA in percentage of total LCPUFA

***
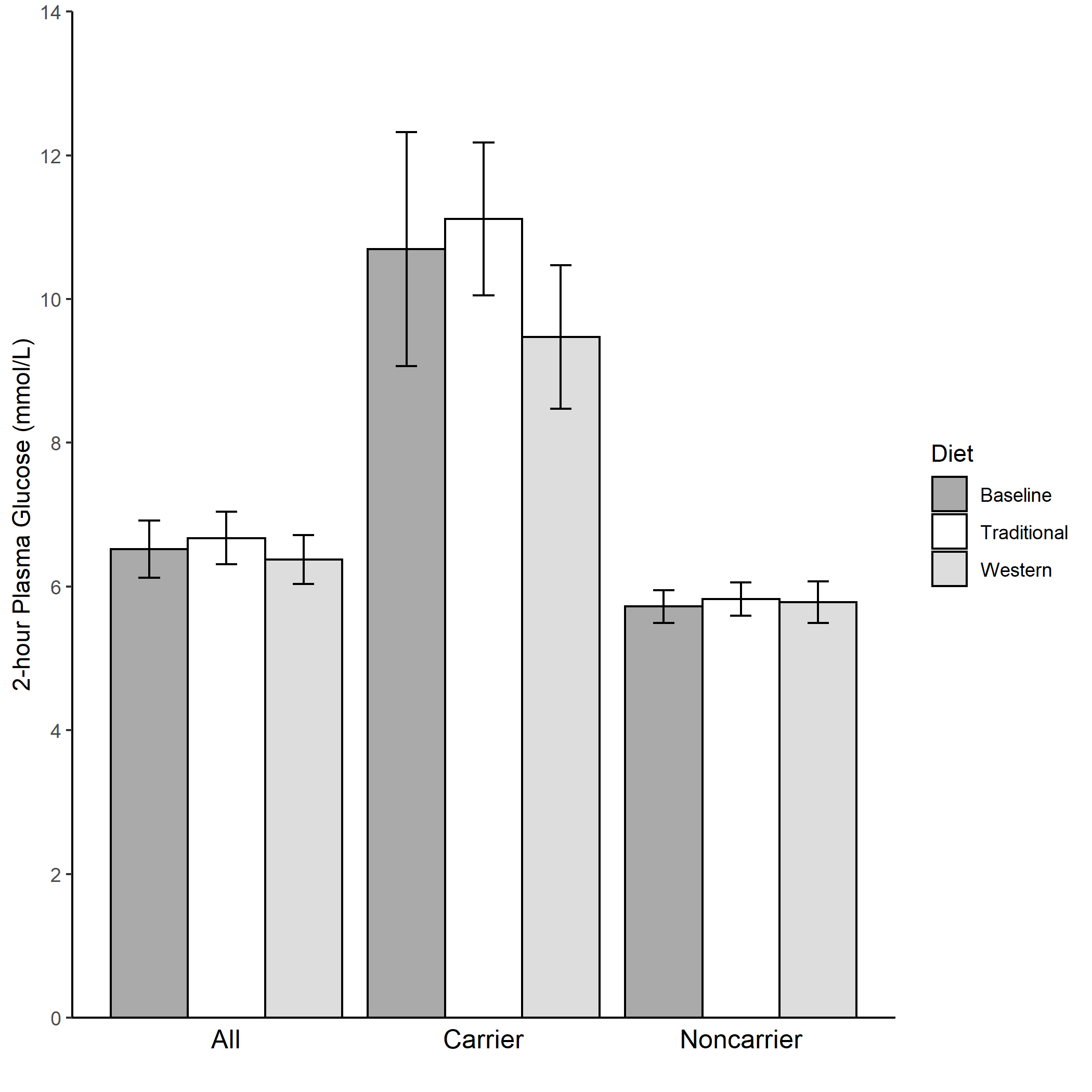
***

**Supplementary Figure 1**. Estimated marginal means and standard error of 2-h plasma glucose derived from two linear mixed models. Bars representing “all” participants derive from a model containing terms for intervention, visit, order, baseline 2h glucose and random effects (site and individual). Bars representing “carrier” and “non-carrier” derive from a model additionally containing a Genotype*Diet interaction term.
